# Are AuPd*TM* (*T* = Sc, Y and *M* = Al, Ga, In), Heusler Compounds Superconductors without Inversion Symmetry?

**DOI:** 10.3390/ma12162580

**Published:** 2019-08-13

**Authors:** Linus Kautzsch, Felix Mende, Gerhard H Fecher, Jürgen Winterlik, Claudia Felser

**Affiliations:** 1 Max Planck Institute for Chemical Physics of Solids, 01187 Dresden, Germany; 2 Johannes Gutenberg—Universität, Institut für Analytische Chemie und Anorganische Chemie, 55122 Mainz, Germany

**Keywords:** Heusler compounds, superconductivity, electronic structure, non-centrosymmetric

## Abstract

Heusler compounds with 2:1:1 stoichiometry either have a centrosymmetric Cu${}_{2}$MnAl structure or an Li${}_{2}$AgSb structure without a centre of inversion. The centrosymmetry is always lost in quaternary Heusler compounds with 1:1:1:1 stoichiometry and LiMgPdSn structure. This presents the possibility of realizing non-centrosymmetric superconductors in the family of Heusler compounds. The objective of this study is to search for and investigate such quaternary derivatives of Heusler compounds, particularly with respect to superconductivity. Several compounds were identified by carrying out calculations from first principles and superconductivity was observed in experiments conducted on AuPdScAl and AuPtScIn at the critical temperatures of 3.0 and 0.96 K, respectively. All investigated compounds had a valence electron count of 27, which is also the case in centrosymmetric Heusler superconductors.

## 1. Introduction

Ternary intermetallic Heusler compounds having the $T_{2}T^{\prime }M$ stoichiometric composition, where *T* is a transition metal, $T^{\prime }$ is a transition metal or rare earth element, and *M* is a main group element, have attracted attention because of the Cu${}_{2}$MnAl archetype [1], which is a remarkable ferromagnet despite the absence of any ferromagnetic element. Nowadays, Heusler compounds are mainly associated with spintronics applications [2,3] as half-metals [4] or spin gapless semiconductors [5]. In half-metals, only electrons with one spin direction exhibit a density of states at the Fermi energy [6], owing to the peculiar exchange splitting of the *d*-electron states.

**Figure 1:**
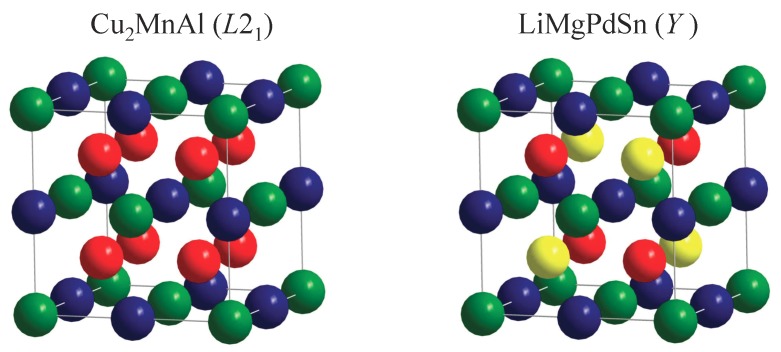
Crystal structures of Heusler compounds with and without centre of inversion. The figure shows sketches of the regular, centrosymmetric Cu${}_{2}$MnAl structure with $O_{h}$ symmetry and the quaternary, non-centrosymmetric LiMgPdSb structure with $T_{d}$ point group symmetry. The accompanying space groups are $F\;m\bar{3}m$ and $F\;\bar{4}3m$, respectively.

To date, several Heusler compounds have been observed to exhibit superconductivity [7], and most of them are based on Pd${}_{2}$, while a few are based on Ni${}_{2}$ or Au${}_{2}$. The first Heusler superconductors were reported in 1982 [8]. Amongst them, Pd${}_{2}$YSn has had the highest recorded critical temperature of 5.5 K [9]. Pd${}_{2}$YbSn and Pd${}_{2}$ErSn both have rare earth metals at the $T^{\prime }$ position, and exhibit the coexistence of superconductivity and antiferromagnetic order [10,11]. Recently, the enhancement of superconductivity through a charge density wave quantum critical point was reported in the (Pt${}_{1-x}$Pd${}_{x}$)${}_{2}$LuIn series of Heusler compounds [12]. The centrosymmetric $L2_{1}$ structure belonging to the space group $F\;m\bar{3}m$ is common in all of these superconducting Heusler compounds (Figure 1). Most of the known superconducting Heusler compounds have a valence electron ($e^{-}$) count of 27 (excluding the eventual 4f electrons) [7]. In previous work, we carried out electronic structure calculations for Heusler compounds to investigate candidates for superconductivity, and identified several Pd${}_{2}$- and Ni${}_{2}$-based Heusler compounds that become superconducting in the low temperature region [13,14,15]. To this end, we used a method that searches for typical fingerprints, such as saddle-point singularities, in the vicinity of the Fermi energy in the band structure of the compounds.

Previously, it was found that most known superconductors with a regular Heusler structure ($L2_{1}$) contain 27 valence electrons corresponding to 6.75$e^{-}$ per atom (see [7] and the references therein). We will return to this point later in the text. Apart from the 27 valence electrons, another fingerprint is the saddle-point-type van Hove singularity [16]. The Bardeen–Cooper–Schrieffer (BCS) theory of superconductivity states that the transition temperature of a BCS superconductor exponentially increases with the increasing density of states n(E) at the Fermi energy $\epsilon_{F}$, provided that the Debye temperature $\theta_{D}$ and Cooper-pairing interaction are independent of $n(\epsilon_{F})$. The saddle points in the energy dispersion curves of the electronic structure of solids lead to shallow maxima in the density of states (DOS). The coincidence of this van Hove singularity with the Fermi energy may cause an enhanced density of states at $\epsilon_{F}$; therefore, it may be a good precondition for superconductivity. Moreover, $\theta_{D}$ may be substituted by the Fermi temperature $\theta_{F}$, which allows higher transition temperatures compared with BCS theory. This so-called van Hove scenario has been considered as an example for explaining the unusually high transition temperatures of the intermetallic A15 superconductors [17], cuprate superconductors [18], and Heusler compounds [15,19].

For certain types of superconductors, the Matthias rule relates the critical temperature ($T_{c}$) to the valence electron count. Matthias reported that the maximum $T_{c}$ of pure elements for a valence electron concentration is slightly below 5$e^{-}$/atom [20], and subsequently proposed the existence of two maxima close to 5 and 7$e^{-}$/atom [21] for binary alloys. It is proposed that Heusler superconductors also follow such an empirical rule, with most superconductors found at 6.75 and 7$e^{-}$/atom [7]. The latter electron concentration results in 28$e^{-}$ per primitive cell.

In fact, there exists a relationship between the Matthias rule and the van Hove scenario. The number of valence electrons determines the band filling, and thus the position of the Fermi energy in the band structure. For compounds with a given structure and stoichiometry, the band structure is similar and only slightly changes with different lattice parameters. For a given electron concentration, this means that the position of the saddle or other singular points in the band structure will not change much with respect to the Fermi energy. However, the different masses of the contributing elements will change the phonon characteristics, and thus the electron-phonon coupling. Moreover, the electron–phonon coupling is disregarded both in the Matthias rule and in the van Hove scenario, which limits the validity of these simple empirical rules. The role of the electron-phonon coupling on the superconducting gap and critical temperature is manifested for example in the Eliashberg theory and the Allen–Dynes formula for $T_{c}$ [22,23] and is discussed in detail in textbooks on superconductivity [24,25].

Most superconductors, including the Heusler compounds, exhibit crystal structures with inversion symmetry. The binary $T_{3}M$ (T= transition metal and M= main group element) compounds that exhibit superconductivity crystallize in the A15 lattice with $O_{h}$ symmetry (space group $P\;m\bar{3}n$). The Pd${}_{2}T^{\prime }M$, Ni${}_{2}T^{\prime }M$, or Au${}_{2}T^{\prime }M$ Heusler compounds are known to be superconducting and also exhibit $O_{h}$ symmetry ($F\;m\bar{3}m$). This also concerns the quaternary, or better known as pseudo-ternary, alloys Pd${}_{2}T_{1-x}^{\prime }T_{x}^{\prime \prime }$Al with $T^{\prime }=$ Zr or Hf, and $T^{\prime \prime }=$ Y or Nb [15]. However, the superconducting state requires a reduction of symmetry, as has already been demonstrated by Cracknell [26]. As a real vector, a current allows for symmetry operations in point group $C_{\infty \;v}$ with the current along the rotational axis. Assuming a supercurrent along *z*, the overall symmetry (crystal structure plus current) must be reduced from $O_{h}$ to $C_{4v}$ or from $T_{d}$ to $C_{2v}$ [27]. Generally, this reduced symmetry does not involve the atomic positions, but rather involves the electron wave functions. (Note that the symmetry of the positions alone does not depend on external fields.) An antisymmetric potential gradient gives rise to a parity-breaking antisymmetric spin–orbit interaction [28]. Hence, there is significant interest in investigating superconducting systems wherein the crystal structure is already non-centrosymmetric. Moreover, non-centrosymmetric superconductors [28] are important with respect to understanding superconducting gap symmetries and pairing mechanisms. The major concern of these superconductors is their pairing state when the inversion centre is lacking. Parity is no longer necessary for the symmetry of the Cooper pairs; therefore, the pairing state should be a mixture of singlet and triplet Cooper pairs. The first report of superconductivity in 2004 in non-centrosymmetric systems, such as the strongly correlated heavy fermion superconductors CePt${}_{3}$Si [29] or CeRhSi${}_{3}$ [30], has raised interest with regard to clarifying the influence exerted by the lack of inversion symmetry on the superconducting properties of compounds.

Heusler compounds are known to crystallize not only in centrosymmetric structures, but also in structures without an inversion centre. Examples include the $C1_{b}$ structure (MgAgAs, cF12, $F\;\bar{4}3m$ (216)) of $TT^{\prime }M$ compounds, and the *X*-type (Li${}_{2}$AgSb) and *Y*-type (LiMgPdSn, cF16, $F\;\bar{4}3m$ (216)) structures. For the quaternaries, mainly the cubic *Y*-type is significant as a structure without an inversion centre. Additionally, this structure is related very closely to the $L2_{1}$ Heusler structure, except for the lack of inversion symmetry, as shown in Figure 1. Further, the *Y*-type structure is also obtained when filling the vacancy of the ternary $C1_{b}$ structure by a fourth, different atom. Moreover, in many cases, the electronic structure of the centrosymmetric Heusler compounds and that of compounds with non-centrosymmetric LiMgPdSn structure are shown to be quite similar.

This study used the same design criterion as previous studies [13,14,15] to search for quaternary Heusler compounds that correspond with the scenarios described above. The starting point was Au- and Pd-based materials that exhibit superconductivity in the $T_{2}T^{\prime }M$ stoichiometry when T= Au or Pd. Using this method, AuPdScAl was identified as a potential candidate for non-centrosymmetric superconductivity. The calculation details, synthesis, structural characterization, and investigation of the superconducting transport properties of AuPdScAl are presented below.

## 2. Materials and Methods

### 2.1. Details of Calculations from First Principles

The electronic structure was calculated using so called full potential (FP) methods within the framework of the local density approximation to the density functional theory. The generalized gradient approximation was used for the exchange-correlation functional in the parametrizations of Perdew, Burke, and Enzerhof [31]. For structural optimizations and basic calculations, the full potential linearized augmented plane wave method was used as implemented in Wien2k (versions 13.1–18.2) [32]. The equilibrium lattice parameters obtained from the volume optimization were slightly larger, but the results for the band structure remained unchanged compared with using the experimental lattice parameters. Owing to the high “Z” of Au, the spin–orbit interaction was included in the calculations.

Further calculations were performed using the fully relativistic, full potential program Sprkkr (version 7.1) [33,34]. In particular, this method was used to calculate the Bloch spectral functions. Calculations for the disordered compounds with mixed site occupations were carried out with Sprkkr using the coherent potential approximation (CPA) in full potential mode. CPA allowed the simulation of a random site occupation using different elements [35]. Additional details regarding the various parameters entering the calculations can be found in References [36,37].

### 2.2. Experimental Details

Polycrystalline ingots of AuPdScAl (and other compounds, as listed in Table A1 of Appendix A) were prepared by the repeated arc melting of the stoichiometric mixtures of the corresponding elements in an argon atmosphere. Caution was exercised to avoid oxygen contamination. An ingot of titanium was melted before the samples as a getter for residual oxygen in the chamber. To compare the sample purity, various samples were subsequently annealed for two weeks at 873 and 1073 K in evacuated quartz tubes. After the annealing process, the samples were quenched in a mixture of ice and water to retain the desired structure.

The cleanness, phase purity, and composition of the samples was investigated using energy dispersive X-ray spectroscopy (EDX) with a JEOL JSM-7800F scanning electron microscope (JEOL, Tokyo, Japan) and a BRUKER XFlash 6130 detector (BRUKER, Billerica, MA, USA). From the EDX investigation, the phase separation of AuPdScAl into the regular Heusler compounds Au${}_{2}$ScAl and Pd${}_{2}$ScAl can be excluded. A very small minority phase (<1%) with a stoichiometry of approximately 1:1:3:1 was observed in several samples. According to the EDX analysis on seven different areas of the sample, the main AuPdScAl phase had in average the composition of 24:26:24:26 (±1%). This is within the expected 1:1:1:1 stoichiometry considering the experimental uncertainty (note that Au and Pd were analyzed at *L* lines and Sc and Al at *K* lines). Inductively coupled plasma optical emission spectrometry (Agilent 5100 ICP-OES, Agilent, Santa Clara, CA, USA) on larger pieces of the sample resulted in the correct 1:1:1:1 composition. The results for other compounds were similar.

The structural and transport properties of the single phase compounds were investigated by conducting detailed experiments to verify the superconducting state. The crystal structure was investigated by powder X-ray diffraction (XRD). The XRD measurements were carried out using Siemens D5000 (BRUKER, Billerica, MA, USA) or Huber G670 (HUBER Diffraktionstechnik GmbH & Co. KG, Rimsting, Germany) diffractometers with monochrome Cu $K_{\alpha }$ radiation. The powder XRD data were processed and analyzed by Powdercell (Federal Institute for Materials Research and Testing (BAM), Berlin, Germany), Fullprof (version 6.1) [38], WinX${}^{\mathrm{POW}}$ (version 1.1) (Stoe and Cie, Darmstadt, Germany), and/or Topas (version 6) (BRUKER, Billerica, MA, USA). The lattice parameter of AuPdScAl was determined to be a=6.442 Å (for others see Appendix A). Again, a phase separation of AuPdScAl into Au${}_{2}$ScAl and Pd${}_{2}$ScAl can be excluded, because the lattice parameters of the regular Heusler compounds differ by 0.2 Å [39] and such a difference can be easily detected by XRD.

The transport properties (resistivity and specific heat) of the single phase samples were measured using a physical property measurement system (PPMS, Quantum Design, San Diego, CA, USA, Model 6000). The magnetic properties were measured using a SQUID magnetometer (Quantum Design, San Diego, CA, USA, MPMS-XL-5).

## 3. Calculation Results

**Figure 2:**
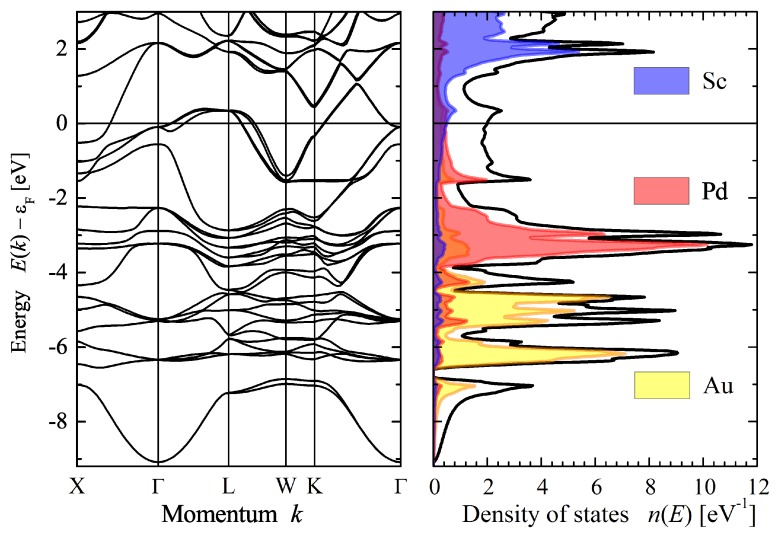
Electronic structure of AuPdScAl. The figure shows the band structure and density of states calculated with Wien2k [32], including the spin–orbit interaction. The total density of states is displayed by the black line.

Here, the electronic structure of AuPdScAl will be discussed. Additional band structures for other compounds can be found in the supplemental material (Figures S1–S8). Figure 2 shows the calculated electronic structure of AuPdScAl. The splitting of *d* bands caused by the spin–orbit interaction is clearly visible, and it is also clear that the *d* states of the transition metals split from each other (Pd from about −3.5 to −2 eV and Au from −6.5 to −4 eV). As expected, the larger splitting was observed for the Au *d* bands. All localized *d* states were further away in energy (below −2 eV) from the Fermi energy, and the bands crossing $\epsilon_{F}$ mainly had a delocalized character. Two van Hove singularities appeared close to the Fermi energy: one at Γ ($T_{d}$ symmetry) and another saddle point at *L* ($C_{3v}$ symmetry). The former was just below $\epsilon_{F}$ but did not significantly contribute to the density of states n(E), owing to the rather free electron character of the bands and the missing degeneracy of the Γ point. The states had a four-fold degenerate $\Gamma_{8}$ character. The other eight-fold degenerate singularity at *L* resulted in the local maximum of n(E) at approximately 350 meV above $\epsilon_{F}$, which is similar to the van Hove singularity observed in the Pd${}_{2}$-based Heusler compounds [15]. Here, the two states with the $L_{4}$ and $L_{5}+L_{6}$ character arose from the splitting (2 meV) of a former *e* state (note: *e* is the two-fold degenerate irreducible representation of the cubic group $T_{d}$).

## 4. Experimental Results

The detailed results obtained by the structural characterization and investigation of the superconducting transport properties are presented below.

### 4.1. X-ray Diffraction

**Figure 3:**
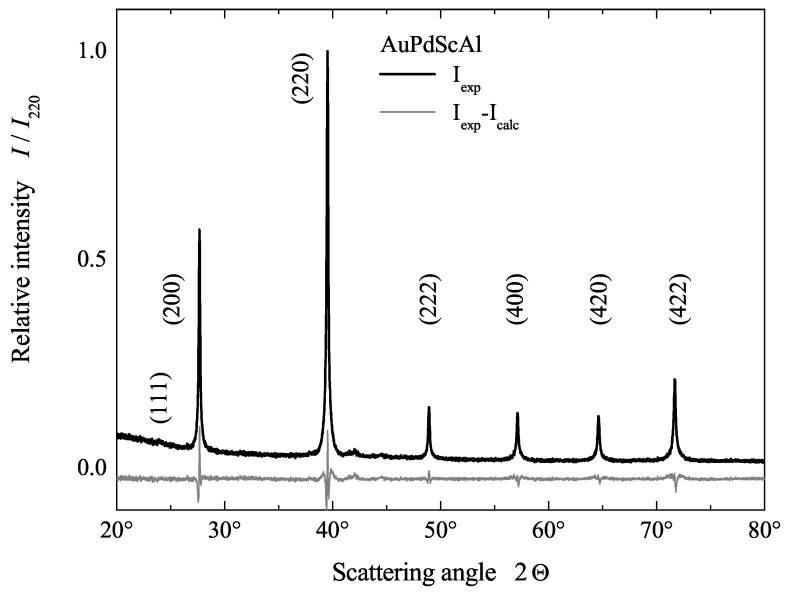
Powder X-ray diffraction of AuPdScAl. The figure shows the observed diffraction data (black). The difference curve (grey) shows the difference between the observed data and the Rietveld refinement. The measurement was performed at room temperature (≈300 K).

Figure 3 shows the x-ray diffraction (XRD) pattern of AuPdScAl. The crystal structure was analyzed in terms of the space group $F\;\bar{4}3m$ (216) with the Wyckoff positions occupied by the atoms as follows: Au at 4d, Pd at 4c, Sc at 4b, and Al at 4a, as shown in Figure 1. The cubic Heusler-type fcc structure can be easily recognized in Figure 3. The pattern indicates the phase purity of the sample. The small feature in the vicinity of the (220) reflection indicates a small impurity amount with 1:1:3:1 stoichiometry. However, the details of the XRD pattern of AuPdScAl reveal that the compound did not crystallize in the well-ordered LiMgPdSn structure. A detailed analysis of the (111), (200), (311), (331), and (511) reflections revealed that there was a high degree of disorder amongst the 4c and 4d sites of the fcc lattice (Pd and Au), which resulted in the pronounced deviation in the abovementioned reflections. This type of antisite disorder created a virtual centre of symmetry which led, on average, to a Cu${}_{2}$MnAl structure with the change of symmetry from $T_{d}$ to $O_{h}$ on the macroscopic scale.

Rietveld refinement was performed with an *R*-value of 3.2% and it confirmed the disorder between the Pd and Au. A lattice parameter of a=6.442 Å was derived from the refinement of AuPdScAl. Anti-site disorder is a common feature in Heusler compounds and, in many cases, the degree can be reduced through annealing procedures. Therefore, the samples were annealed at 873 and 1073 K. These temperatures were useful in improving the structural order of the reported Pd${}_{2}$-based Heusler superconductors [15]. The XRD patterns of both samples did not exhibit any visible improvement with regard to the disorder between the Pd and Au atoms. The influence of the disorder on the electronic structure will be discussed in detail in Section 5.1.

### 4.2. Electric Resistance

**Figure 4:**
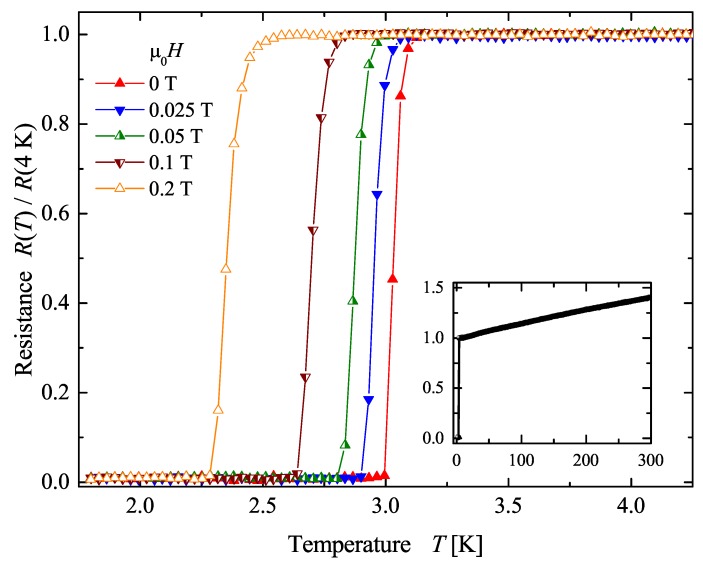
Resistance of AuPdScAl. The figure shows the temperature dependence of the resistance in the $T_{c}$ region, at five different magnetic induction fields $\mu_{0}H$. The inset shows the resistance of AuPdScAl for an expanded temperature range.

Temperature dependent resistance measurements were carried out in different magnetic fields to demonstrate the superconductivity of the compound. Samples with polished surfaces were analyzed using the four point probe technique. Figure 4 shows the resistance of AuPdScAl as a function of temperature at five different magnetic fields. The superconducting transition was observed at approximately 3.0 K in the field free case (0.96 K for AuPtScIn). With increasing magnetic fields, $T_{c}$ shifted to lower temperatures. $T_{c}=2.3$ K was observed at a magnetic induction field of 0.2 T. For magnetic induction fields of 0.5 T and above, superconducting transition was not observed above 1.8 K. The characteristic of the resistance was metallic at high temperatures, as can be seen in the inset. The residual resistance ratio R(300K)/R(4K) of approximately 1.5 is a common value for polycrystalline bulk intermetallic samples, and is comparable to known values for this class of disordered Heusler compounds. The low value of R(300K)/R(4K) arises from chemical disorder scattering that influences in alloys the resistivity more than the electron-phonon scattering. With a value of below 5 K, the transition temperature was in the range of those reported for other Heusler compounds [7,9].

### 4.3. Magnetic Properties

**Figure 5:**
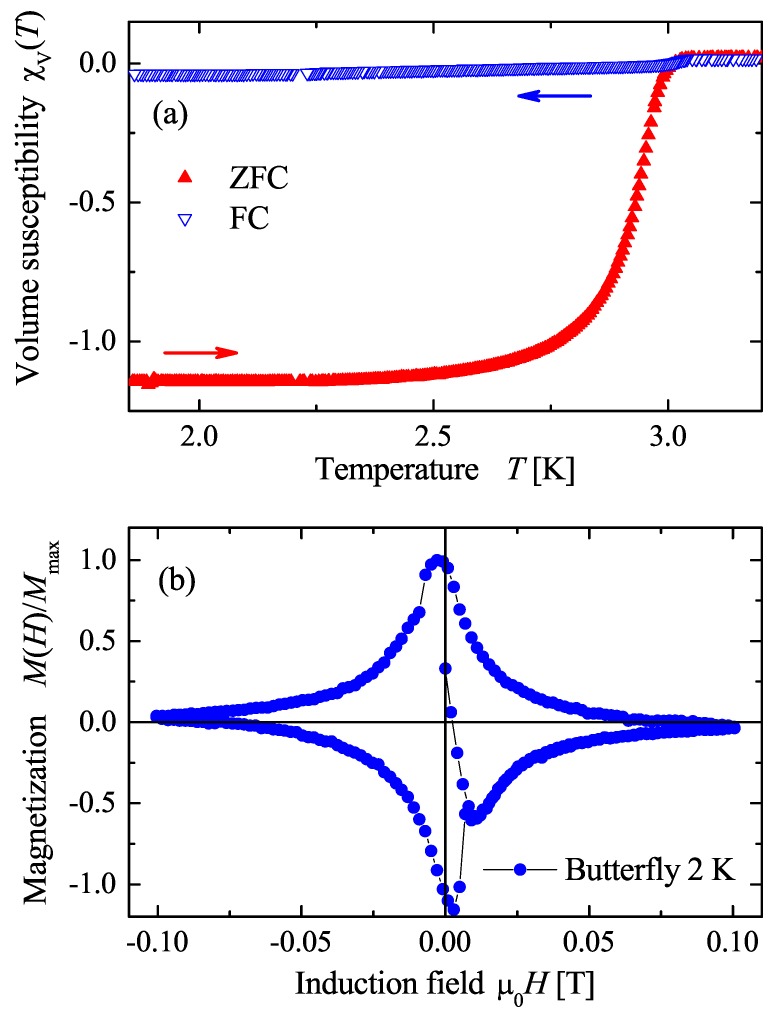
Diamagnetic shielding and Meissner effect for AuPdScAl. Panel (**a**) shows the temperature dependence of the susceptibility in a magnetic induction field of 2.5 mT under zero field cooled (ZFC) and field cooled (FC) conditions. Panel (**b**) shows the butterfly loop for AuPdScAl at 2 K.

Magnetization measurements were carried out to investigate the diamagnetic shielding and Meissner effect in AuPdScAl bulk samples. The results of the magnetization measurements are shown in Figure 5. The upper panel (a) shows the temperature dependent magnetic volume susceptibility $\chi_{V}\left(T\right)$ of the sample in an external induction field of 2.5 mT. For the calculation of $\chi_{V}\left(T\right)$, a demagnetization factor of 1/3 for a spherical sample geometry was assumed. The zero-field cooled (ZFC) curve shows the diamagnetic shielding of AuPdScAl. The onset of the superconducting transition was observed at the critical temperature of $T_{c}\approx 3$ K. The sharpness of the transition indicates good sample purity and quality. The demagnetization factor used provided a bulk superconductivity of approximately 115% for the investigated sample. Notably, the demagnetization factor of 1/3 may be too low because the shape of the sample was not exactly spherical. The field cooled (FC) curve reflects the Meissner effect in AuPdScAl. The large difference in magnitude between the ZFC and the FC measurements indicates a comparatively weak Meissner effect and suggests that AuPdScAl is a type II superconductor. This fact is attributed to strong flux pinning in the bulk material.

The lower panel (b) shows the field dependent magnetization (butterfly loop) of AuPdScAl between −100 mT and 100 mT at a temperature of 2 K. At this temperature, the accurate determination of the critical magnetic field $H_{c1}$ is unlikely, owing to the broadening of the magnetization maximum. Additionally, flux pinning causes a more symmetric shape.

Wernick et al. found in susceptibility measurements on the sister compound AuPdYIn a critical temperature of 2.3 K [9]. Unfortunately, they did not perform a structural analysis. We found a $T_{c}$ of 2.62 K for AuPdYIn from the magnetization measurements (see Appendix A for structure data). However, a phase transition could not be observed in the measurement of the specific heat.

### 4.4. Specific Heat

**Figure 6:**
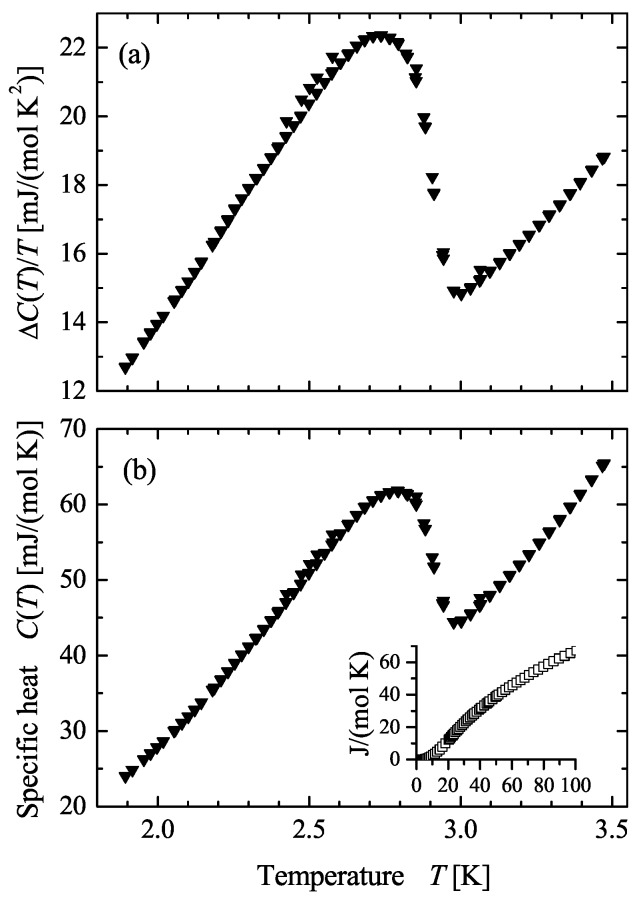
Specific heat of AuPdScAl: (**a**) specific heat divided by temperature (C/T); (**b**) temperature dependent specific heat C(T) on the temperature scale in the region of superconducting transition; the inset shows C(T) in a larger temperature range.

The specific heat of AuPdScAl was measured to investigate its thermal properties. Figure 6 shows the specific heat of AuPdScAl in the temperature region of the superconducting transition. The onset of superconductivity was observed at a temperature of approximately 3 K, according to the electrical measurement shown in Figure 4. The jump of the specific heat at the critical temperature exhibits a certain amount of rounding and broadening, and these curve distortions are attributed to the bulk inhomogeneities of the polycrystalline samples.

## 5. Discussion

### 5.1. Electronic Structure Revisited

The XRD investigations—not only on AuPdScAl—revealed that most of the compounds adopt a seemingly centrosymmetric $L2_{1}$ structure, rather than the non-centrosymmetric *Y*-type structure (see also Appendix A). The co-existence of Au${}_{2}$ScAl and Pd${}_{2}$ScAl can be excluded by EDX and XRD (see Section 2.2). Specifically, this is the average long range property, where the Au and Pd atoms occupy the same crystallographic site at random, and are thus no longer distinguishable by XRD. On the nanoscale, we do not simply have Au${}_{2}$ScAl or Pd${}_{2}$ScAl, but also AuPdScAl regions. Therefore, on average, the inversion centre can be retained only over a large part of the crystal; however, it is still absent in the nanoscale regions, at least. In the regular, primitive fcc cell of the LiMgPdSb structure, there exists exactly one atom per each kind, and any change of the atom positions can maintain the symmetry. In the conventional cubic cell there are 4 Au and 4 Pd atoms (Figure 1). In the case of random distribution, the smallest cubic cell Au:Pd ratios of 4:4, 3:5, 5:3, 2:6, 6:2, 1:7, and 7:1 are observed, but none of these ratios make the cell automatically centrosymmetric, and typically, the local symmetry is further decreased. In fact, the ratios of 8:0 and 0:8, which belong to the regular Cu${}_{2}$MnAl structure, may result in an inversion centre, but not necessarily because of the possible alternative Li${}_{2}$AgSb-type arrangement of the atoms. The same is true if larger cells are used (note that the overall Au:Pd-ratio of 1:1 must always be retained in the macroscopic scale; that is, on average, each cell with an x:y ratio should be accompanied by a cell with an y:x ratio). Thus, it can be safely concluded that the inversion centre observed by XRD was only virtual.

**Figure 7:**
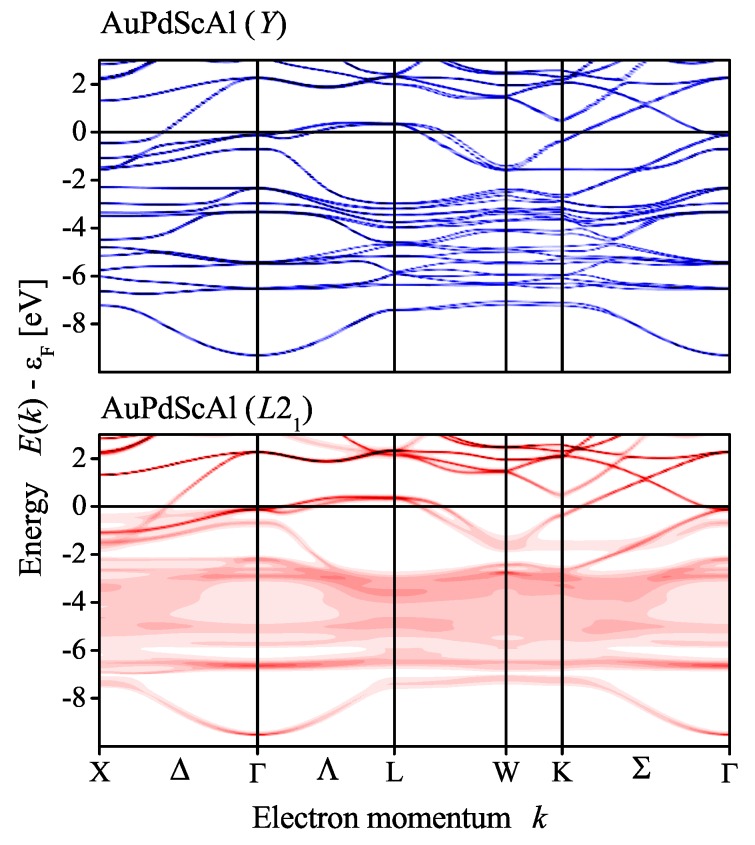
Bloch spectral function of ordered and disordered AuPdScAl.

**Figure 8:**
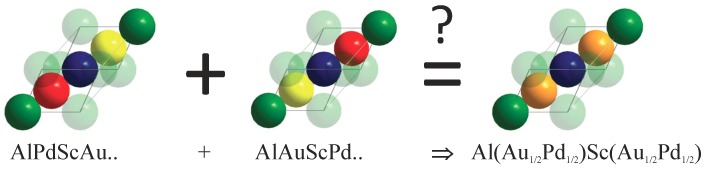
Coherent potential approximation (CPA) scheme from ordered to disordered AuPdScAl along (111) direction. The series of atoms corresponds to the arrangement of atoms along the [111] direction of the primitive fcc cell, which consists of the Wyckoff positions 4a (0,0,0), 4c (1/4,1/4,1/4), 4b (1/2,1/2,1/2), and 4d (3/4,3/4,3/4) of space group $F\;\bar{4}3m$. (also compare Figure 1).

The coherent potential approximation (CPA) was used to investigate the effect of a completely random Au/Pd distribution in the electronic structure, with respect to the mean field. Figure 7 compares the Bloch spectral functions for structures *Y* and $L2_{1}$ by assuming that chemical disorder occurs only between Au and Pd, while disregarding all other types of disorder that may be vacancies or anti-site disorder including Sc and Al. In particular, the 4d and 4c positions of the LiMgPdSn structure were occupied by 50% Au and 50% Pd, which resulted in the proposed virtual Cu${}_{2}$MnAl structure. The scheme is shown in Figure 8. The atomic distribution of the two possible atom arrangements in the primitive fcc cell of the *Y* structure with $T_{d}$ symmetry were averaged such that the resulting structure appeared like $L2_{1}$ with $O_{h}$ point group symmetry. Accordingly, the potential was averaged selfconsistently in the CPA. The question mark at the equality sign serves as a reminder for the fact that the inversion symmetry is lost only in the macroscopic scale, as observed by XRD.

As a result of the chemical disorder, several bands emerging from the Au and Pd *d* electrons became strongly broadened when the two different structures were compared. The broadening mainly occurred for the Au and Pd *d* states; however, the main features close to the Fermi energy were conserved. In particular, the van Hove singularities at Γ and *L* were not destroyed. Notably, the Weyl point appearing in the Δ direction of the $L2_{1}$ structure, just below the Fermi energy, appeared as an avoided crossing in the *Y* structure.

**Figure 9:**
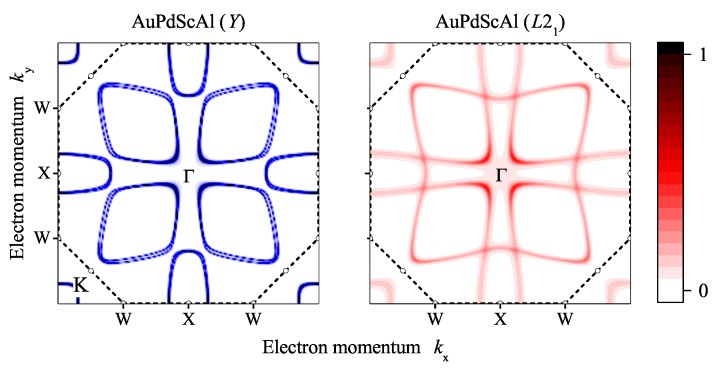
Fermi surface of ordered and disordered AuPdScAl. Comparison of Bloch spectral functions in the xy plane of AuPdScAl in the *Y* and $L2_{1}$ structures. The border of the first Brillouin zone is indicated by the dashed line with open symbols at the positions of the high symmetry points. The colour scale bar is normalized to unity.

Figure 9 compares the cuts through the Fermi surface in the xy plane. Similar to the band structure, the main features are the same in the *Y* and $L2_{1}$ structures. The main difference is the crossing of the bands appearing in the $L2_{1}$ structure, which is avoided in the *Y* structure.

The cuts through the Fermi surfaces were used to calculate the autocorrelation function of the Fermi surface, which is sometimes called the “nesting function” and is defined as follows for well-ordered compounds:

(1) $$f\left(q\right)=\sum \delta \left(E\left(k\right)-\epsilon_{F}\right)\delta \left(E\left(k+q\right)-\epsilon_{F}\right)$$

The nesting function f(q) is related to the imaginary part of the static susceptibility $\chi {}^{\prime \prime }(q,\omega =0)$, as follows:

(2) $$f\left(q\right)=\lim_{\omega \to 0}\frac{\chi {}^{\prime \prime }(q,\omega )}{\pi \omega })$$

In case of disordered compounds with random site occupations, the energy eigenvalues are no longer sharply defined. The chemical disorder scattering results in the broadening of the dispersion curves, as shown in Figure 9. Here, the autocorrelation function is calculated from the self-convolution of the Bloch spectral function at the Fermi energy, as follows:

(3) $$a^{2}\left(q\right)={\left.\int n(E,k)n(E,k+q)dk\right|}_{E=\epsilon_{F}}$$

where n(E,k) is the Bloch spectral function at the wave vector *k*, and n(E,k) is calculated from the imaginary part of the multiple scattering Green’s function and may be interpreted as the local density of states in the *k* space. In practical cases, the Bloch spectral function is given on a discrete *k* mesh and the integration is replaced by a summation similar to Equation (1) with n(k) replacing the δ functions. The autocorrelation function $a^{2}\left(q\right)$ becomes large whenever the wave vector *q* connects two points belonging to the flat extended portions of the Fermi surface, or if *q* connects small regions with a very high density of states, such as van Hove singularities. The former effect is typically termed Fermi surface nesting. In fact, the self-convolution of the Bloch spectral function (Equation (3)) may also be used for well-ordered compounds.

**Figure 10:**
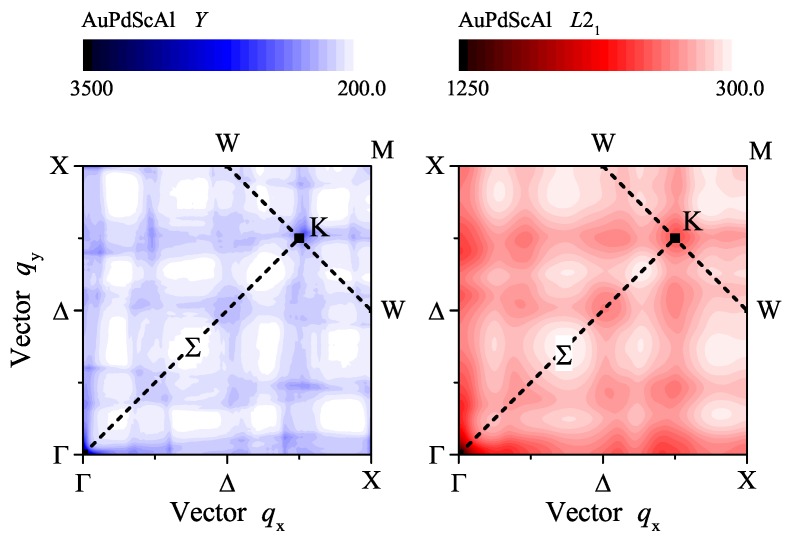
Nesting vectors in ordered and disordered AuPdScAl. The autocorrelation functions ($a^{2}\left(q\right)$) of the Fermi surfaces in the (001) plane of ordered and disordered AuPdScAl are compared for the *Y* and $L2_{1}$ structures. The high symmetry lines and points in the irreducible wedge of the first Brillouin zone are indicated. The scale of the coloured bars is in atomic units.

Figure 10 shows the autocorrelation function ($a^{2}\left(q\right)$) of the Fermi surfaces in the (001) plane of the ordered and disordered AuPdScAl. The autocorrelation functions exhibit a typical, albeit physically meaningless, 1/|q| divergence at the Γ point, and $a^{2}\left(q\right)$ reflects the cubic symmetry of the crystal structures. Various maxima appeared in the autocorrelation function. Most strikingly, the maximum close to the *K* point is present in both situations, independent on the disorder. Specifically, it appeared at $q_{110}^{Y}=1.09\pi /a$ and $q_{110}^{L2_{1}}=1.07\;\pi /a$ for the ordered and disordered structure, respectively. This corresponds to a nesting vector of approximately $\left(\sqrt{1/2},\sqrt{1/2},0\right)$. With a slight variation in the band filling (valence electron concentration), the nesting vector will coincide with the *K* point. This situation may have forced the appearance of a charge density wave as a trigger for the super conductivity, as has been reported for the (Pt${}_{1-x}$Pd${}_{x}$)${}_{2}$LuIn series of Heusler compounds [12].

## 6. Conclusions

This study investigated the structural, magnetic, and electronic properties of AuPdTM and related compounds, with a special emphasis on AuPdScAl. Here, *T* and *M* are the transition and main group elements from groups 3 and 13, respectively. The XRD investigation revealed that most compounds of the series crystallize in a disordered centrosymmetric $L2_{1}$ structure, rather than in an ordered *Y* structure (Table S1). In some cases, such as in AuPtScIn or AuPdYIn, both structures are hardly distinguishable because their constituents have similar atomic scattering factors.

However, the inversion symmetry observed in XRD results from averaging over a large part of the crystal. In the nanoscale regions, the inversion symmetry is absent owing to the random Au:Pd ratios in the different cells. The broadening of the bands based on the chemical disorder only occurs in the Au and Pd *d* states, and both singularities at Γ and *L* remain intact. Therefore, the van Hove scenario is still conserved along with the Matthias rule. Moreover, a nesting vector of approximately $\left(\sqrt{1/2},\sqrt{1/2},0\right)$ was found in the autocorrelation function of the Fermi surface of AuPdScAl.

The resistance and susceptibility measurements of the AuPdScAl compound exhibited a transition into the superconducting state at approximately 3.0 K. The strong flux pinning in the bulk material provided evidence that AuPdScAl is a type II superconductor. Additionally, the transition into the superconducting state was proved by the temperature dependent specific heat C(T).

In conclusion, it was demonstrated by the AuPdTM set of compounds that the prediction of superconductivity by the Matthias rule and van Hove scenario can be extended to quaternary Heusler compounds. These simple models are restricted because they do not include all ingredients determining the critical temperature. For example, phonons and electron-phonon coupling are not considered, as mentioned in the introduction. Thus, some compounds can perhaps feature critical temperatures below the measured temperature range. The ordering on the 4d and 4c positions played a minor role; therefore, the predictions may be unified over ternary and quaternary compounds. Finally, the AuPdScAl, AuPdYIn, and AuPtScIn compounds are promising for further investigations regarding the relationship between superconductivity and nanoscale ordering.

## Supplementary Materials

The following are available online at https://www.mdpi.com/1996-1944/12/16/2580/s1, Figure S1–S8: Electronic structure of the ordered and disordered compounds: AuPdScAl, AuPdScGa, AuPdScIn, AuPdYAl, AuPdYIn, AuPtScIn, AgPdScAl, and CuNiScAl; Table S1: Structural data for the calculated compounds.

## Appendix A. Search for Superconducting Heusler Compounds without Inversion Symmetry

Apart from those already mentioned, additional Heusler compounds were investigated. First, ab initio calculations were performed for compounds that have not been previously reported. Hence, Wien2k (see Section 2.1) was used to determine the optimized lattice constant *a* for various quaternary Heusler compounds with 27 valence electrons, under the assumption of an ordered *Y* structure.

From the compounds with $27e^{-}$ and $T_{1}T_{2}T_{3}M$ composition ($T_{1}=$ Cu, Ag, Au, $T_{2}=$ Ni, Pd, Pt, $T_{3}=$ Sc, Y, La, and M= Al, Ga, In), 19 compounds were synthesized and characterized as described in Section 2.2 (see [40]). Table A1 summarizes the results for the structural data obtained by the experiments and compares them to the theoretical lattice parameters. The latter were calculated for a well-ordered *Y*-type structure. Note that for CuNi-, AgPd-, or AuPt-based compounds, the *Y* and $L2_{1}$ structures were hardly distinguishable, owing to the approximately equal x-ray scattering amplitudes of the neighbouring elements.

**Table A1:** Structural data. The compounds in *italics* were either not synthesized in this study or could not be obtained in a state wherein the characterization of their properties would be reliable; $a_{opt}^{Y}$ are theoretical lattice parameters optimized for the *Y*-type structure, and $a_{XRD}$ are experimental lattice parameters (all lattice parameters are in Å). *n.o.* means that $T_{c}$ could not be observed. Remarks: * The *Y* and $L2_{1}$ structures cannot be distinguished, and the Rietveld refinements result in the same *R*-values; ** only the $L2_{1}$ structure is reported when *Y* was possible but could not be distinguished by XRD; *** a cubic phase was identified in the multi-phased samples, and the tabulated lattice parameter belong to that phase; **** the $L2_{1}$ (6.5989 Å) and *Y* (6.6007 Å) structures had very similar *R*-values. ***** A superconducting transition temperature of 2.3 K was reported in Reference [9].

Compound	$a_{\mathrm{opt}}^{Y}$	$a_{\mathrm{XRD}}$	Structure	$T_{c}$
CuNiScAl	6.1447	6.1233	$\left(Y,L2_{1}\right)$ *	*n.o.*
*CuNiScGa*	6.1316	6.05	[40] **	
*CuNiScIn*	6.3549	6.30	[40] **	
*CuPtScIn*	6.5321	6.46	multi-phase ***	
*AgNiScAl*	6.3578	6.52	[40] **	
*AgNiYAl*	6.6086	——	non-cubic	
AgPdScAl	6.4981	6.4357	$\left(Y,L2_{1}\right)$ *	*n.o.*
*AgPdScIn*	6.6822	6.58	[40] **	
*AgPdYAl*	6.7394	6.7119	multi-phase ***	
*AgPdYIn*	6.8986	6.79	[40] **	
AuPdScAl	6.4907	6.4298	$L2_{1}$	3.0 K
*AuPdScGa*	6.5054	6.43	multi-phase ***	
AuPdScIn	6.6925	6.5989	$L2_{1}$ ****	*****
*AuPdYAl*	6.7341	——	non-cubic	
AuPdYIn	6.9052	6.8137	$L2_{1}$	*n.o.*
*AuPdLaIn*	7.1345	——	non-cubic	
*AuPtScAl*	6.4988	6.51	multi-phase ***	
AuPtScIn	6.6978	6.6011	$\left(Y,L2_{1}\right)$ *	0.96 K
*AuPtLaIn*	7.1312	——	non-cubic	

Four of the compounds exhibited *Y*- or $L2_{1}$-type structures, but a transition into the superconducting state was not observed. One reason might be that $T_{C}$ was too low to be detected in the experiments conducted in this study. These compounds were assigned by $T_{c}$ n.o. in Table A1. Transport measurements were only performed for single phase samples.
