# Supplementary material for: Are AuPdTM (T = Sc, Y and M = Al, Ga, In), Heusler Compounds Superconductors without Inversion Symmetry?
Source: Materials (Basel). 2019 Aug 13;12(16):2580. doi: 10.3390/ma12162580 (PMC6719038; doi:10.3390/ma12162580)
Supplement: Supplementary file 1 [file materials-12-02580-s001.zip › supplement/PdAuTM_supplement.pdf]

Materials **2019**, *xx*, 1–4; doi:10.3390/maxx010005

Supplementary Materials: Are AuPdTM (T = Sc, Y and M = Al, Ga, In) Heusler compounds superconductors without inversion symmetry ?

Linus Kautzsch [1], Felix Mende [1], Gerhard H Fecher [1*], Jürgen Winterlik [2], and Claudia Felser [1]
Add

1. Prolog

This supplement is dedicated to results from ab-initio calculations for the compounds listed in Table S1. The calculations were performed using the fully relativistic, full potential program SPRKKR [1,2]. Calculated and compared are the Bloch spectral functions for different, ordered and disordered structures.

Table S1. Structural data for calculations.
a^Y_{opt} are theoretical lattice parameters optimized for the Y-type structure and a_{XRD} are experimental lattice parameters (all lattice parameters are in Å). $\overline{Z} = \frac{1}{4}\sum_{i=1}^{4} Z_i$ is the average core charge of the compound.

compound	$\overline{Z}$	a^Y_{opt}	a_{XRD}	calculated structures
AuPdScAl	39.75	6.4907	6.4298	Y, $L2_1$, $B2$
AuPdScGa	44.25	6.5054	6.43	Y, $L2_1$
AuPdScIn	48.75	6.6925	6.5989	Y, $L2_1$
AuPdYAl	44.25	6.7341	——	Y, $L2_1$
AuPdYIn	53.25	6.9052	6.8137	Y, $L2_1$
AuPtScIn	56.75	6.6978	6.6011	Y, $L2_1$
AgPdScAl	31.75	6.4981	6.4357	Y, $L2_1$
CuNiScAl	22.75	6.1447	6.1233	Y, $L2_1$

2. Electronic structure of selected, quaternary Heusler compounds.

For all listed compounds, the electronic structures of the Y and $L2_1$ type structures are compared. For AuPdScAl, two additional disordered structures are respected, that are a 75:25 mixture of the Y and $L2_1$ structures as well as the $B2$ structure. In the later case, Au and Pd are occupying randomly the 1b and Sc and Al randomly the 1a site of a CsCl type structure with space group $Pm\overline{3}m$. To be compatible to the other structures, the set-up was realised on the sites of the Y structure, that is site 1a is simulated by 4a+4b and 1b by 4c+4d, both with 50:50 occupation by the different atoms.

The resulting Bloch spectral functions are shown in the following. It is found that they are all very similar independent on the compound. No strong difference is observed between the compounds with lowest (CuNiScAl) and highest (AuPtYIn) average core charge. The heavier elements result in an only moderate spin orbit splitting of the band compared to the lighter elements. The disorder in the $L2_1$ structure leads to a noticeable broadening of the low lying d bands. However, the bands close to the Fermi energy are not much effected and their principal character, shape, and energetic positions are kept.

2.1. *AuPd based compounds.*

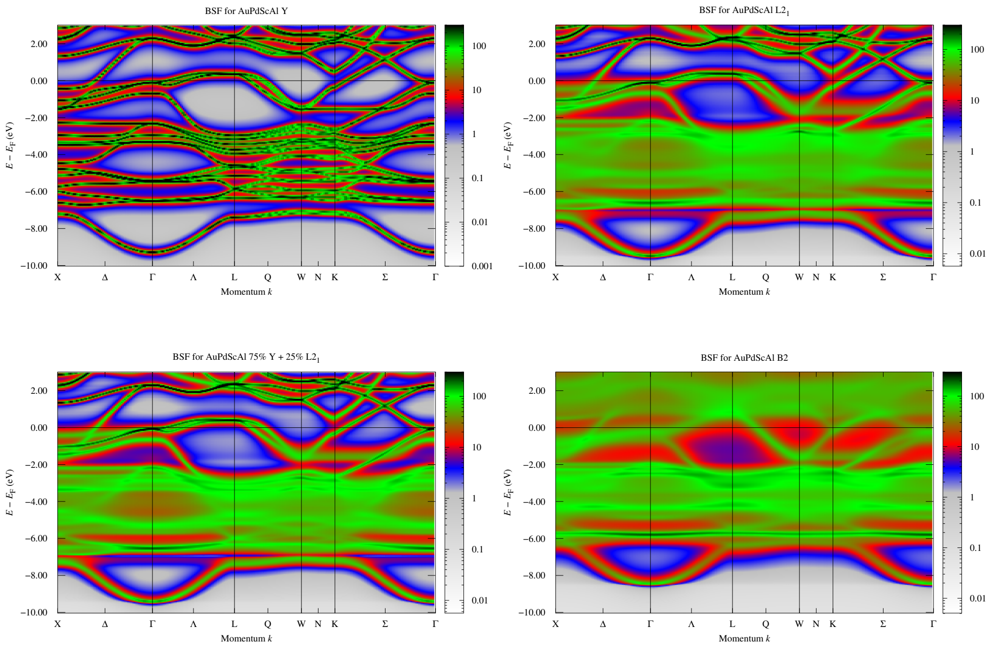

Figure S1. Electronic structure of ordered and disordered AuPdScAl.
Shown are, on top from left to right the Bloch spectral functions for the ordered Y and fully disordered $L2_1$ structures, on bottom from left to right those for the partially disordered $Y + L2_1$, and fully disordered $B2$ structures. All plots are for the fcc Brillouin zone. The colour bars represent the local density of states in atomic units.

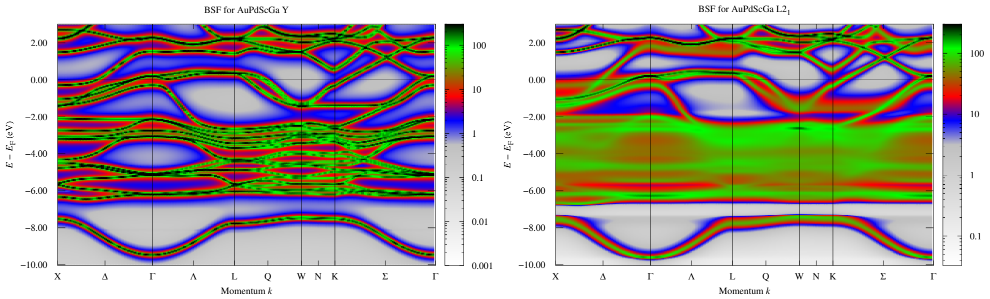

Figure S2. Electronic structure of ordered and disordered AuPdScGa.
Shown are, from left to right, the Bloch spectral functions for the Y and $L2_1$ structures.

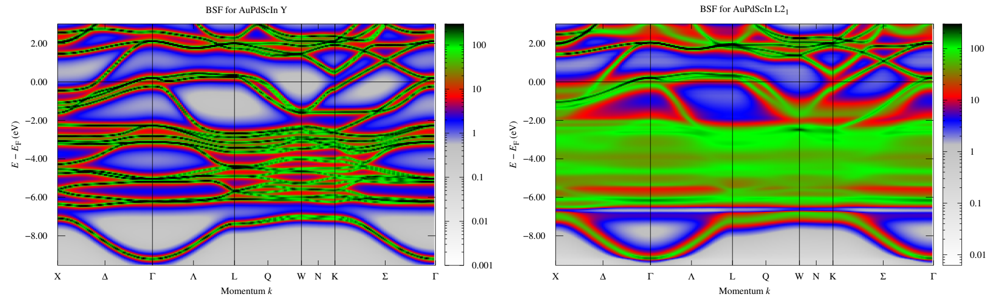

Figure S3. Electronic structure of ordered and disordered AuPdScIn.

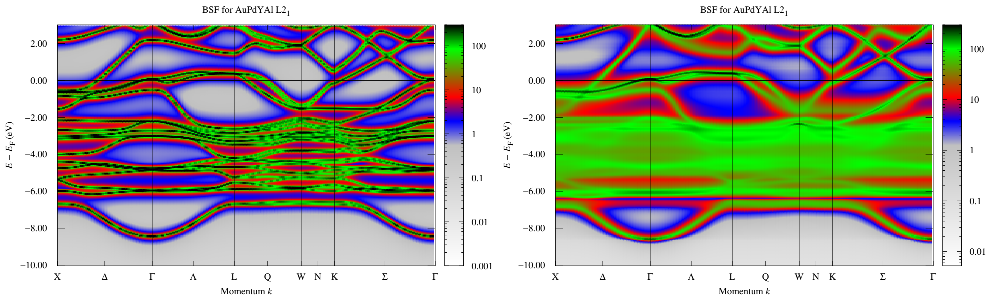

Figure S4. Electronic structure of ordered and disordered AuPdYAl.

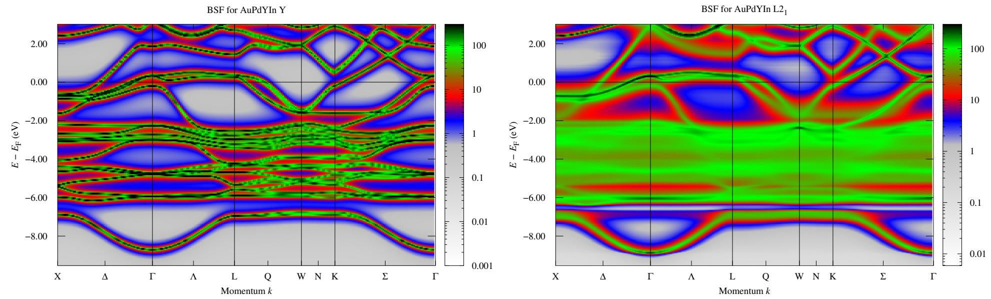

Figure S5. Electronic structure of ordered and disordered AuPdYIn.

2.2. *An AuPt based compound.*

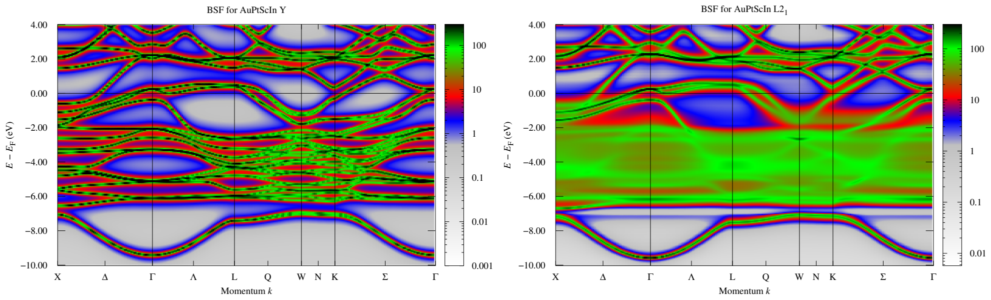

Figure S6. Electronic structure of ordered and disordered AuPtScIn.

2.3. An Ag based compound.

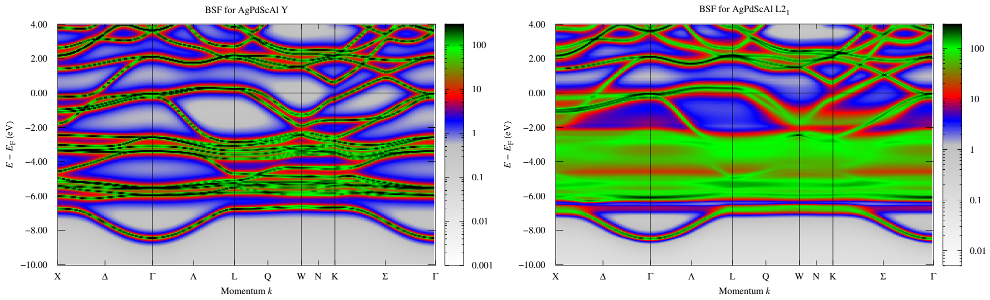

Figure S7. Electronic structure of ordered and disordered AgPdScAl.

2.4. A Cu based compound.

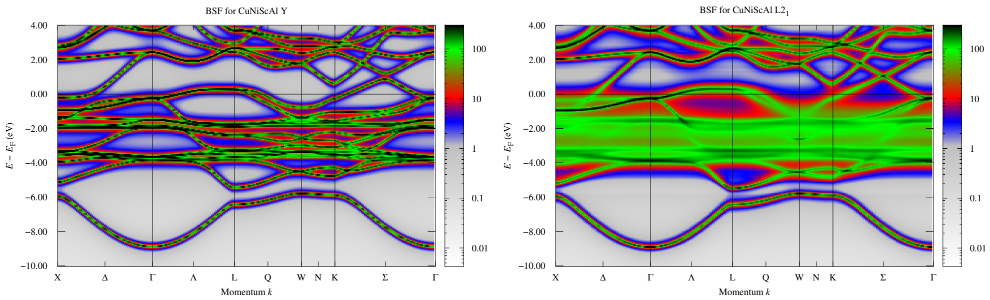

Figure S8. Electronic structure of ordered and disordered CuNiScAl.

References

1. Ebert, H., Fully Relativistic Band Structure Calculations for Magnetic Solids - Formalism and Application. In *Electronic Structure and Physical Properties of Solids. The Use of the LMTO Method*; Dreysse, H., Ed.; Springer-Verlag: Berlin, Heidelberg, 1999; Vol. 535, *Lecture Notes in Physics*, p. 191.
2. Ebert, H.; Ködderitzsch, D.; Minar, J. *Rep. Prog. Phys* **2011**, *74*, 096501.
