# Supplementary figures and images for: Are AuPdTM (T = Sc, Y and M = Al, Ga, In), Heusler Compounds Superconductors without Inversion Symmetry?
Source: Materials (Basel). 2019 Aug 13;12(16):2580. doi: 10.3390/ma12162580 (PMC6719038; doi:10.3390/ma12162580)

BSF for AgPdScAl L2<sub>1</sub>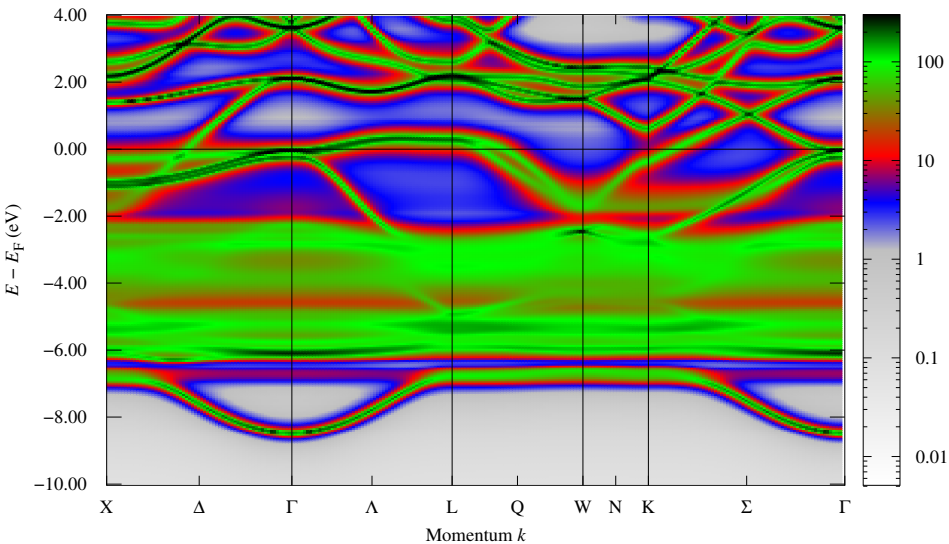

Supplement: Supplementary file 1 [file materials-12-02580-s001.zip › supplement/AgPdScAl_BLOCHSF_L21-eps-converted-to.pdf]

BSF for AgPdScAl Y

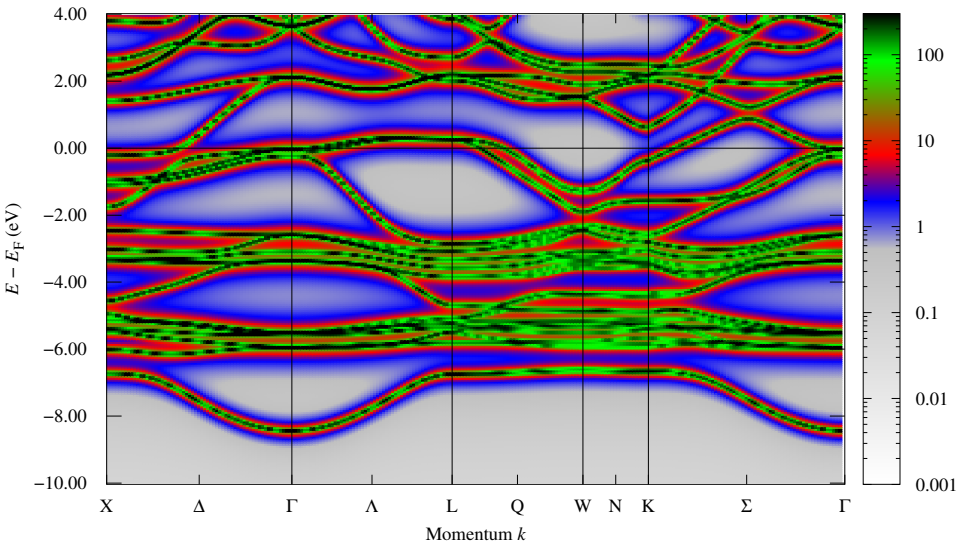

Supplement: Supplementary file 1 [file materials-12-02580-s001.zip › supplement/AgPdScAl_BLOCHSF_Y-eps-converted-to.pdf]

BSF for AuPdScAl B2

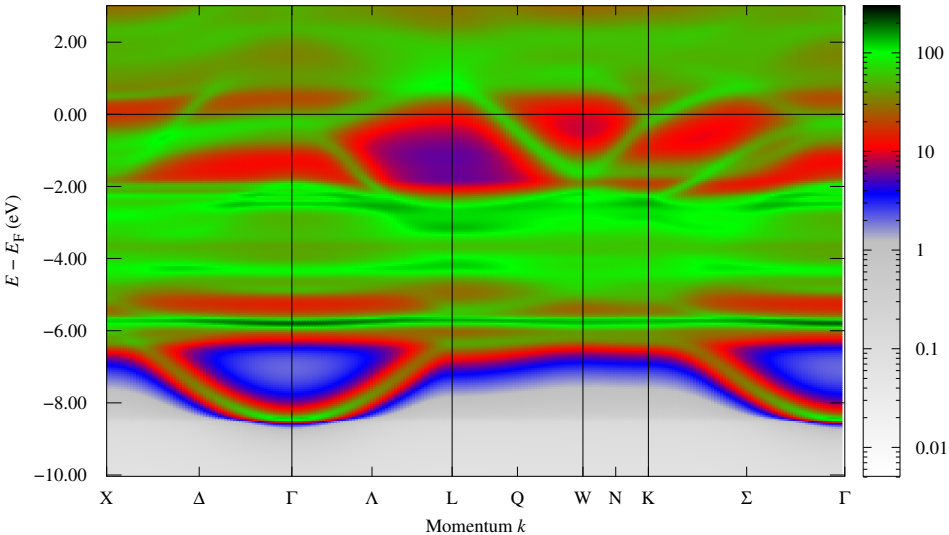

Supplement: Supplementary file 1 [file materials-12-02580-s001.zip › supplement/AuPdScAl_BLOCHSF_B2-eps-converted-to.pdf]

BSF for AuPdScAl L2<sub>1</sub>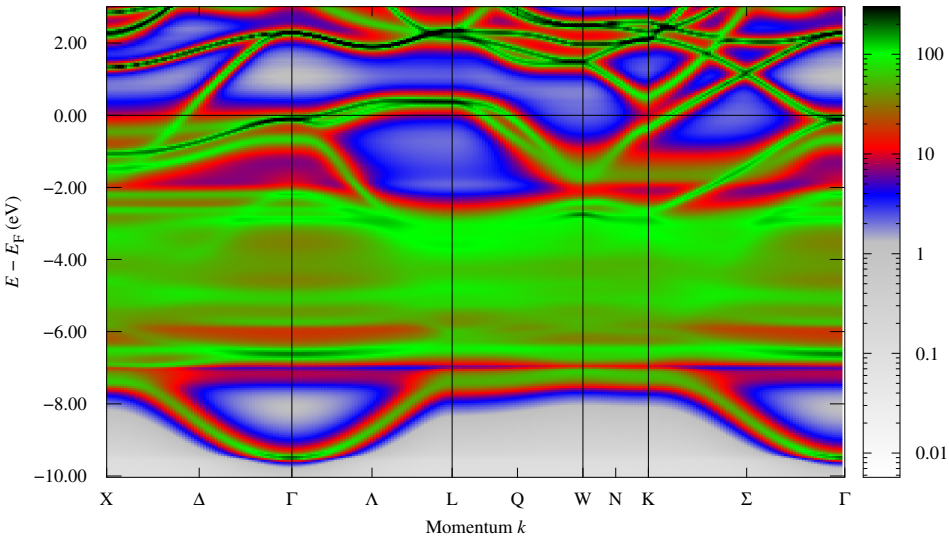

Supplement: Supplementary file 1 [file materials-12-02580-s001.zip › supplement/AuPdScAl_BLOCHSF_L21-eps-converted-to.pdf]

BSF for AuPdScAl Y

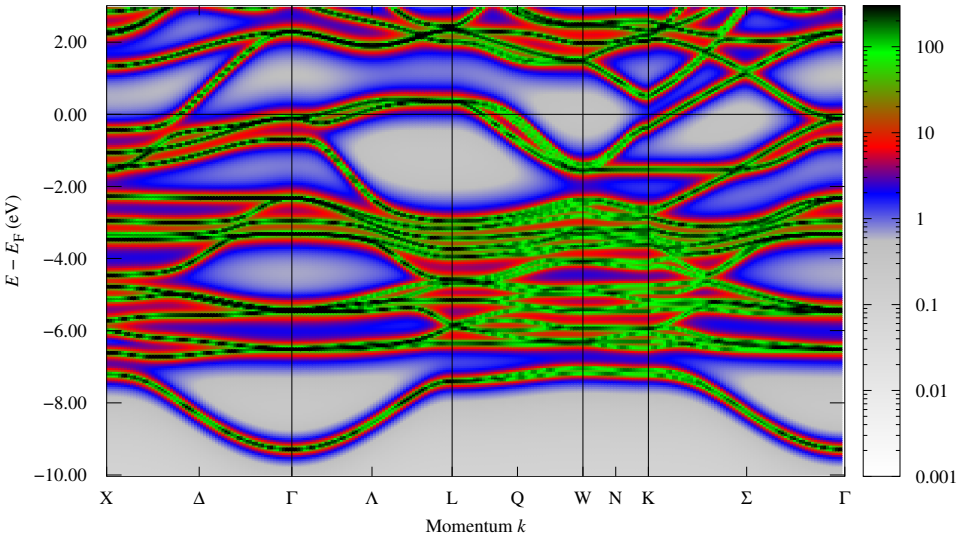

Supplement: Supplementary file 1 [file materials-12-02580-s001.zip › supplement/AuPdScAl_BLOCHSF_Y-eps-converted-to.pdf]

BSF for AuPdScAl 75% Y + 25% L2<sub>1</sub>

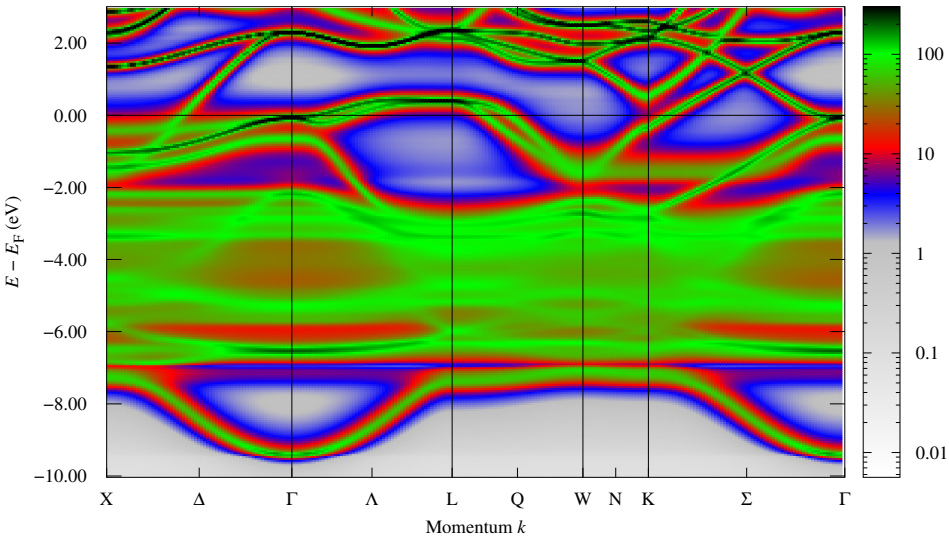

Supplement: Supplementary file 1 [file materials-12-02580-s001.zip › supplement/AuPdScAl_BLOCHSF_Y_75-25-eps-converted-to.pdf]

BSF for AuPdScGa L2<sub>1</sub>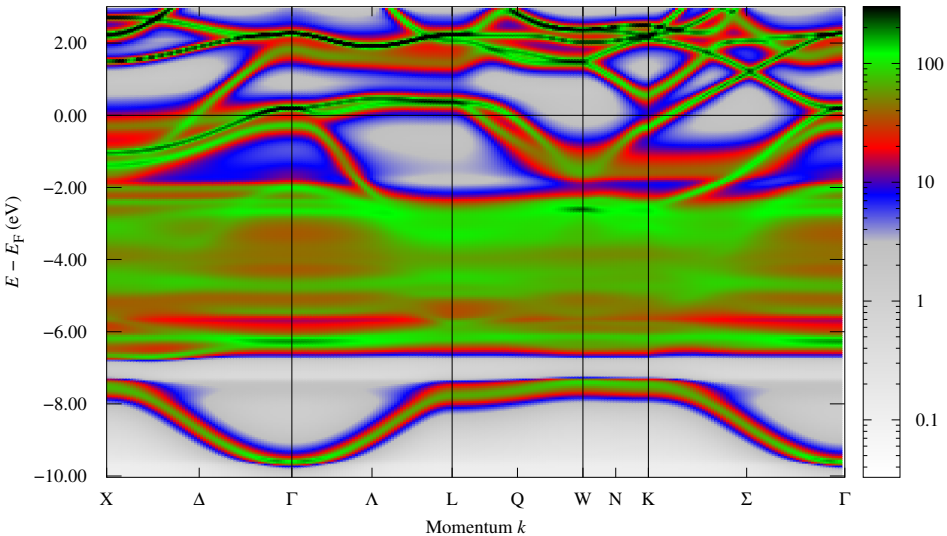

Supplement: Supplementary file 1 [file materials-12-02580-s001.zip › supplement/AuPdScGa_BLOCHSF_L21-eps-converted-to.pdf]

BSF for AuPdScGa Y

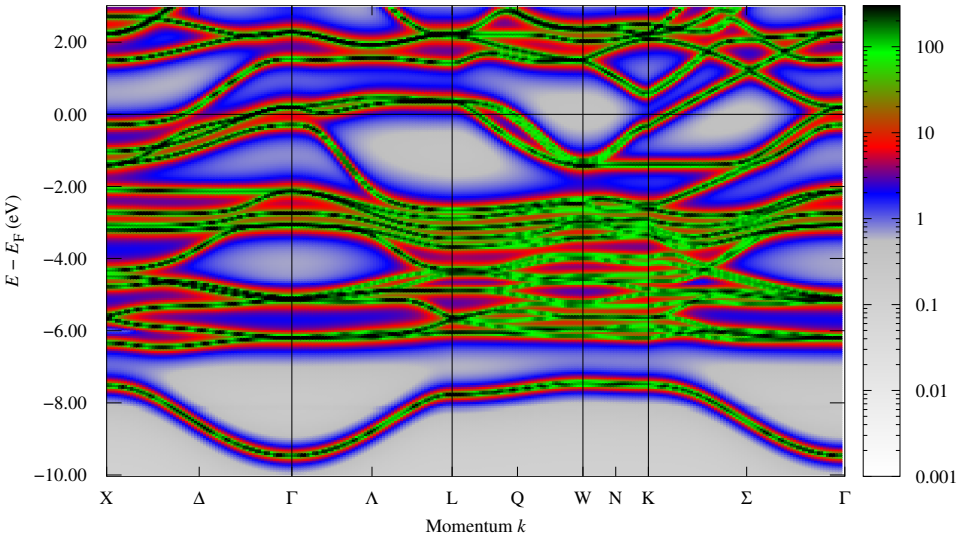

Supplement: Supplementary file 1 [file materials-12-02580-s001.zip › supplement/AuPdScGa_BLOCHSF_Y-eps-converted-to.pdf]

BSF for AuPdScIn L2<sub>1</sub>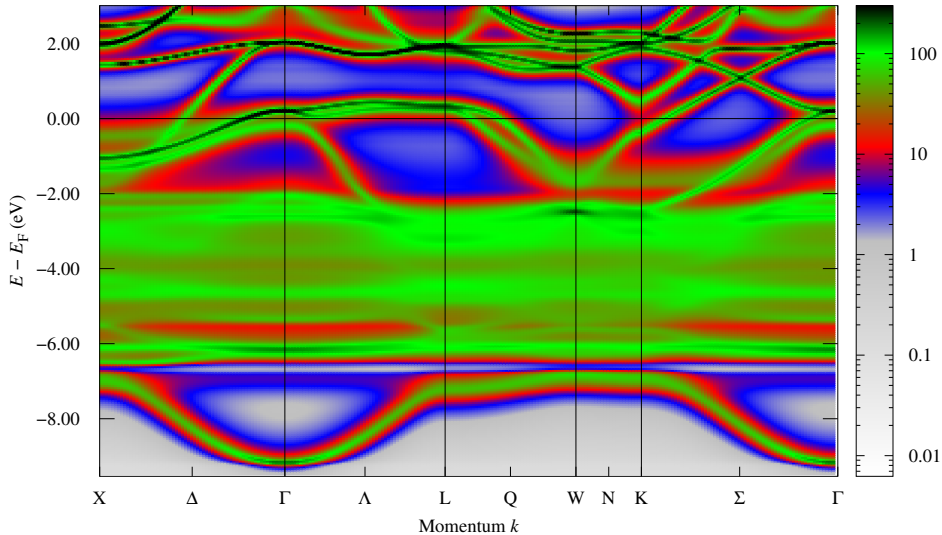

Supplement: Supplementary file 1 [file materials-12-02580-s001.zip › supplement/AuPdScIn_BLOCHSF_L21-eps-converted-to.pdf]

BSF for AuPdScIn Y

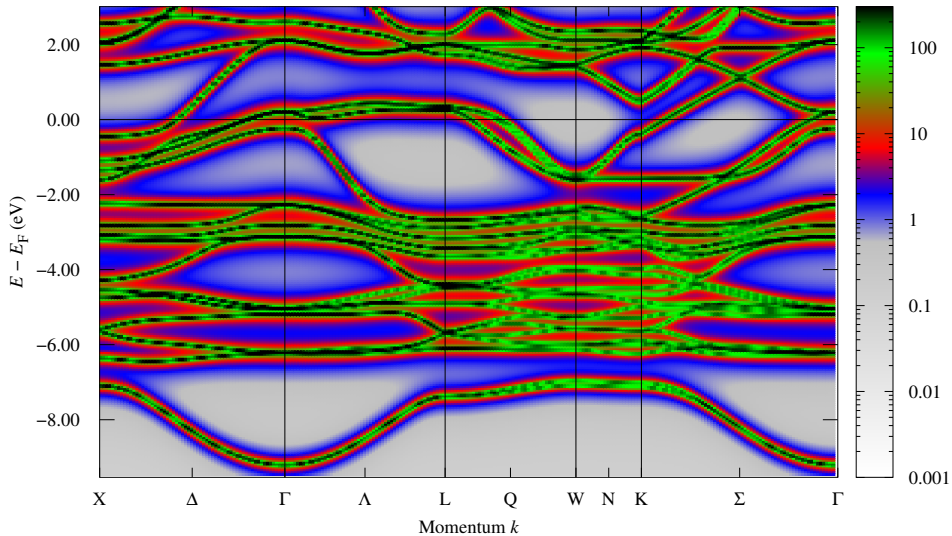

Supplement: Supplementary file 1 [file materials-12-02580-s001.zip › supplement/AuPdScIn_BLOCHSF_Y-eps-converted-to.pdf]

BSF for AuPdYAl L2<sub>1</sub>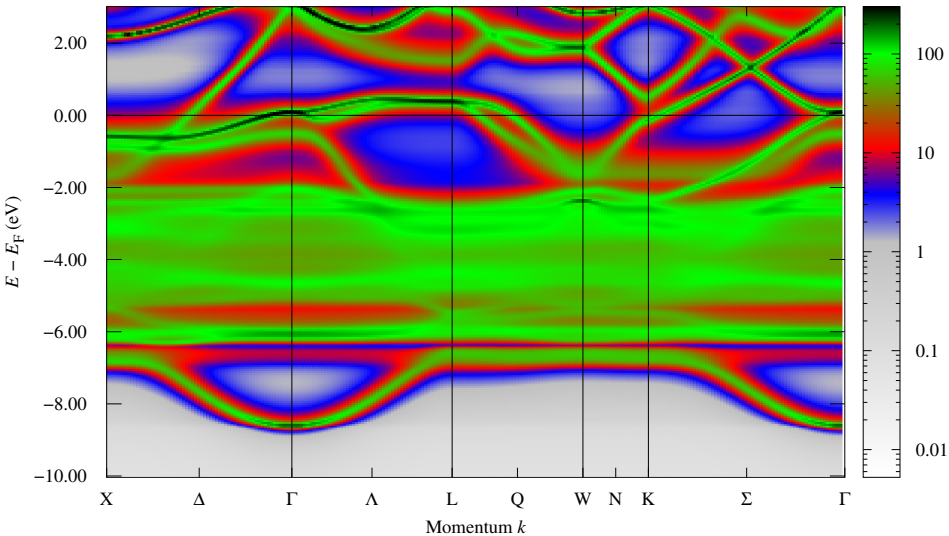

Supplement: Supplementary file 1 [file materials-12-02580-s001.zip › supplement/AuPdYAl_BLOCHSF_L21-eps-converted-to.pdf]

BSF for AuPdYAl L2<sub>1</sub>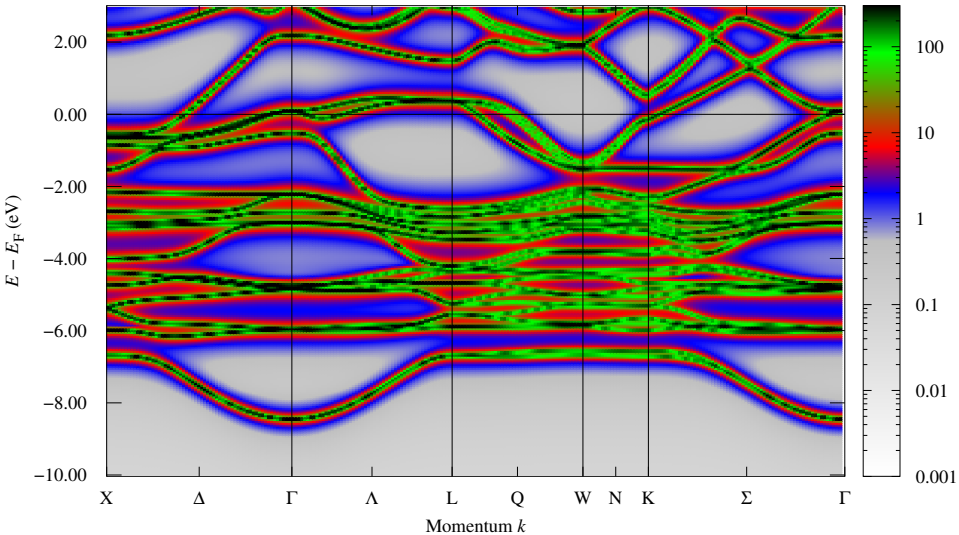

Supplement: Supplementary file 1 [file materials-12-02580-s001.zip › supplement/AuPdYAl_BLOCHSF_Y-eps-converted-to.pdf]

BSF for AuPdYIn L2<sub>1</sub>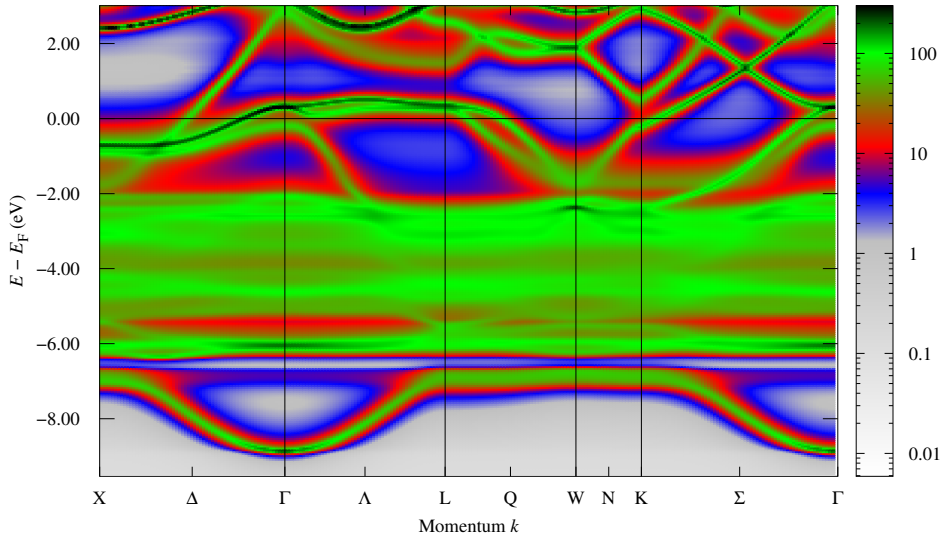

Supplement: Supplementary file 1 [file materials-12-02580-s001.zip › supplement/AuPdYIn_BLOCHSF_L21-eps-converted-to.pdf]

BSF for AuPdYIn Y

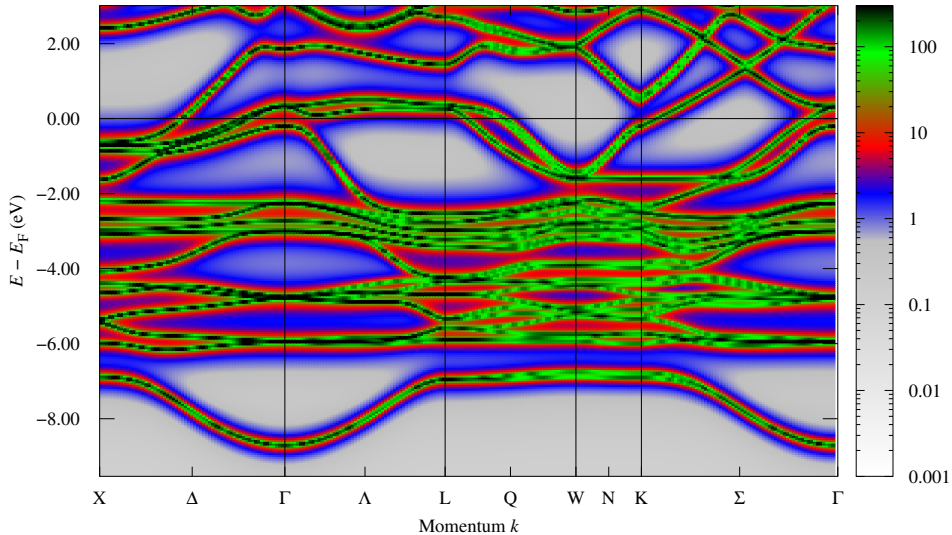

Supplement: Supplementary file 1 [file materials-12-02580-s001.zip › supplement/AuPdYIn_BLOCHSF_Y-eps-converted-to.pdf]

BSF for AuPtScIn L2<sub>1</sub>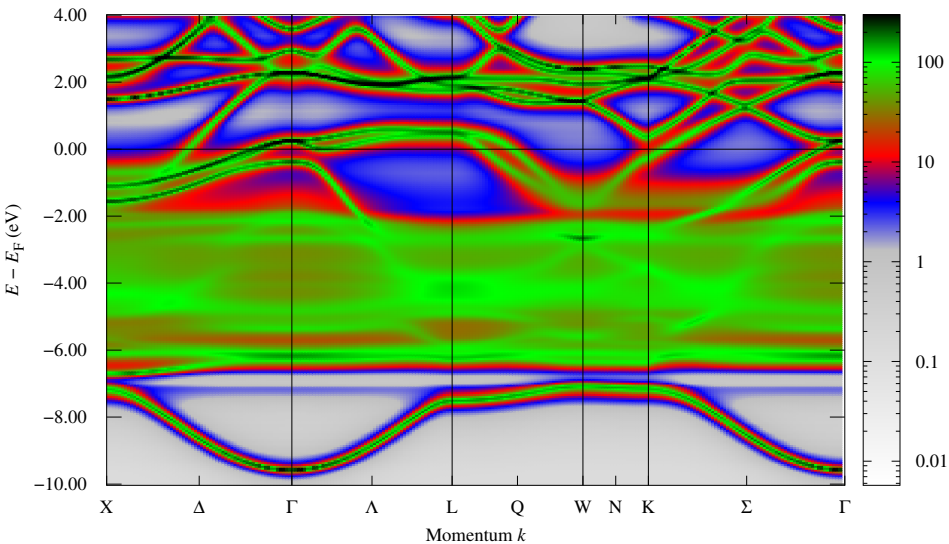

Supplement: Supplementary file 1 [file materials-12-02580-s001.zip › supplement/AuPtScIn_BLOCHSF_L21-eps-converted-to.pdf]

BSF for AuPtScIn Y

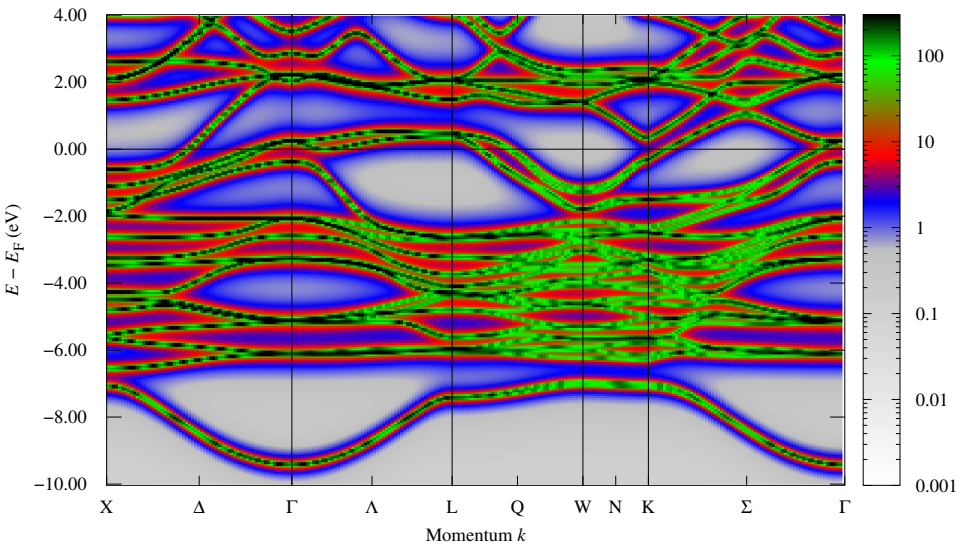

Supplement: Supplementary file 1 [file materials-12-02580-s001.zip › supplement/AuPtScIn_BLOCHSF_Y-eps-converted-to.pdf]

BSF for CuNiScAl L2<sub>1</sub>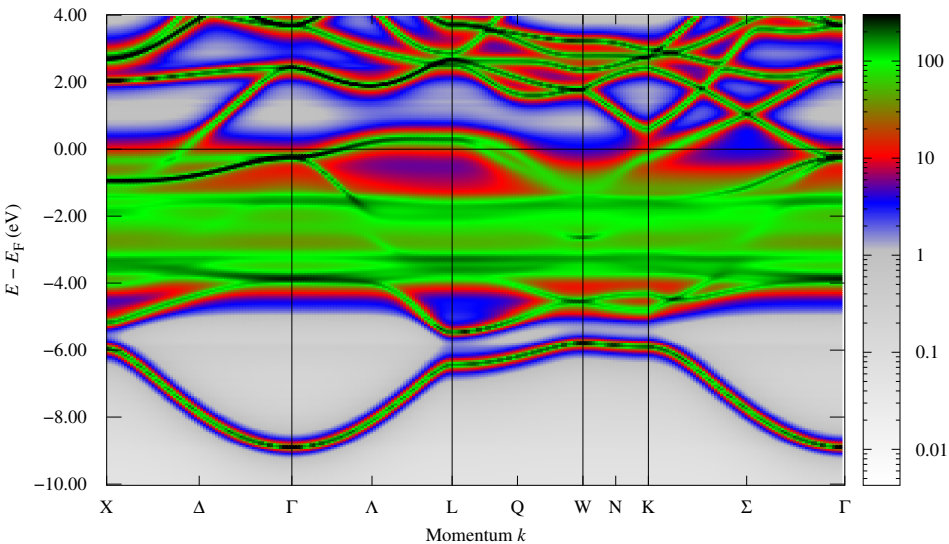

Supplement: Supplementary file 1 [file materials-12-02580-s001.zip › supplement/CuNiScAl_BLOCHSF_L21-eps-converted-to.pdf]

BSF for CuNiScAl Y

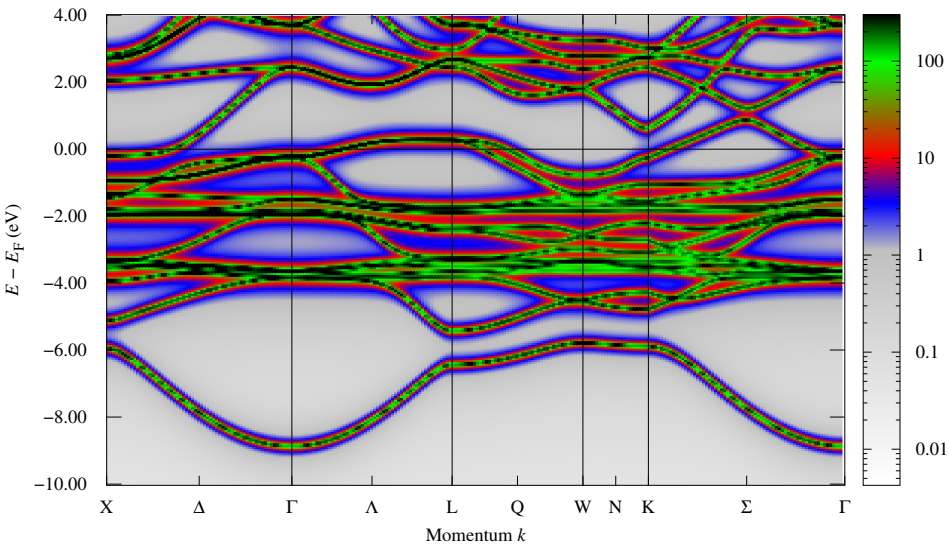

Supplement: Supplementary file 1 [file materials-12-02580-s001.zip › supplement/CuNiScAl_BLOCHSF_Y-eps-converted-to.pdf]

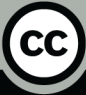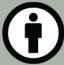

BY

Supplement: Supplementary file 1 [file materials-12-02580-s001.zip › supplement/Definitions/logo-ccby-eps-converted-to.pdf]

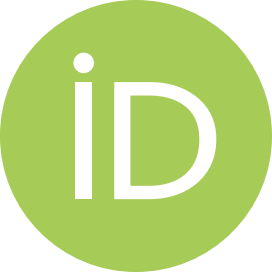

Supplement: Supplementary file 1 [file materials-12-02580-s001.zip › supplement/Definitions/logo-orcid-eps-converted-to.pdf]

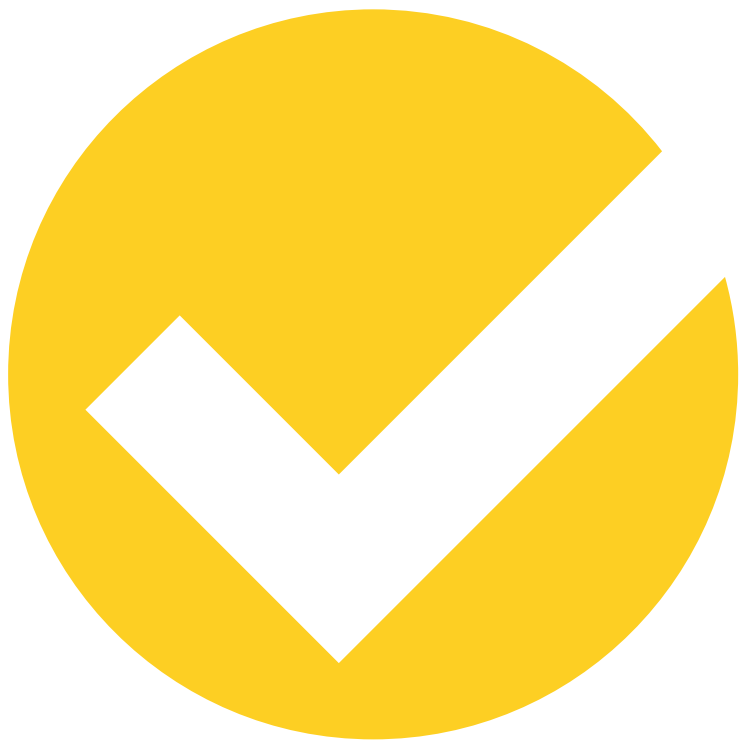

check for  
updates

Supplement: Supplementary file 1 [file materials-12-02580-s001.zip › supplement/Definitions/logo-updates.pdf]

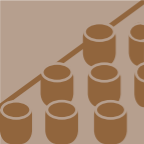

*materials*

Supplement: Supplementary file 1 [file materials-12-02580-s001.zip › supplement/Definitions/materials-logo-eps-converted-to.pdf]
